# Rationale for Accreditation Criteria for the MATES in Construction Workplace Suicide Prevention Program

**DOI:** 10.1177/10482911261449730

**Published:** 2026-05-26

**Authors:** Anthony D. LaMontagne, Tania King, Jorgen Gullestrup, Tu Boldeman, Nicholas Thompson, Brad Parker, Karyn Dale, Liam Cubbage, Trent Bazley, John Chapman

**Affiliations:** 1 Institute for Health Transformation, 110577 Deakin University, Burwood, Australia; 2 Melbourne School of Population and Global Health, University of Melbourne, Melbourne, Australia; 3 Social Equity Research Centre, School of Global Urban Social Studies, 50066 RMIT University, Melbourne, Australia; 4 649105 MATES in Construction, Brisbane, Australia; 5 649105 MATES in Construction, Sydney, Australia; 6 649106 MATES in Construction, Adelaide, Australia; 7 649105 MATES in Construction, Perth, Australia; 8 MATES in Construction, Auckland, New Zealand

**Keywords:** Suicide, construction, blue-collar, accreditation, intervention, prevention

## Abstract

MATES in Construction originated in Australia as a workplace suicide prevention program for predominantly male, blue-collar industries with high suicide rates. Accreditation criteria were developed for the purpose of providing recognition for participating sites and promoting the program. Arising from an internal review of the MATES accreditation criteria, this article presents the purpose and history of MATE's accreditation criteria; the rationale and evidence underpinning the accreditation criteria; and the evolving practice of accreditation in the MATES context.

Four accreditation criteria are specified, addressing the 3 levels of the prevention and early treatment spectrum for mental health and illness interventions (universal/selective/indicated): (1) All the available workforce on a work site must be offered General Awareness Training (GAT, universal); (2) Following initial GAT training, at least 80% of the workforce is GAT trained (universal); (3) All workers must have a “line of sight” to a worker/volunteer trained in how to connect people to help (selective); (4) the work site must be able to access an Applied Suicide Intervention Skills Trained person during operational hours (indicated).

MATES programs continue to evolve, possibly warranting additional accreditation criteria in the future to acknowledge the implementation of increasingly comprehensive programs.

The extent to which the MATES justification for and approach to accreditation is generalizable to other contexts is not known; however, the underlying principles and rationale provided are likely adaptable in other contexts.

## Introduction

MATES in Construction^1^ is a workplace suicide prevention program established by the construction industry in Australia in response to elevated suicide rates among workers in the construction sector^2,3^—historically up to double the rate of other working males.^4,5^ The MATES program is “industry-based” meaning that the program is deliberately placed and seen by workers in the industry as a bi-partisan construction (union and employer) industry institution belonging to the industry. Importantly MATES is seen as being “by the industry for the industry.”^6^

The elevated suicide rates among construction workers, both in Australia and internationally, are driven by a range of factors.^5,7,8^ The construction context is often characterized by high job insecurity, long working hours, high prevalence of bullying and harassment, long commuting times, distant work, and other mentally adverse working conditions, many of which increase suicide risk.^9^ In addition, the workforce is predominantly male. Maladaptive traditional male cultures discourage help-seeking and the discussion of emotions. They often support alcohol and other drugs as acceptable coping mechanisms for stress.

In response to the reports of elevated suicide rates in the construction industry, the Building Employees Redundancy Trust (BERT) funded and established the MATES in Construction program. MATES is jointly owned and controlled by BERT, unions, employers, and employer associations in the industry. The program was cooperatively co-designed by these stakeholders and runs as an independent charity, thus constituting a neutral ground for employers, unions, and workers (as opposed to a union- or employer-based program).

Further, the workforce, contractors, and subcontractors often change over the course of a project. The industry basis of the program is intended to make the benefits of the program transferable between jobs and ultimately to lead culture change in the industry through the organization's four pillars of raising awareness, building capacity, providing help, and conducting ongoing research.^1^ While the program is industry-based, its delivery is site-focused, aiming to reach all workers on a given project or job site; this is why “accreditation,” as detailed below, is site-based.

MATES began operation in Australia in 2008 in the construction industry. The program has since expanded to the mining, energy and manufacturing industries and, geographically, to New Zealand. To date, the MATES programs have reached over 427,300 workers across all industries in Australia and New Zealand. Most recently, MATES is engaged in a pilot project in the North American construction industry.

Accreditation criteria were developed by the MATES organization for the purposes of providing recognition to participating sites and of promoting the dissemination and adoption of the MATES program. The posting and public display of MATES accreditation at the gates of a construction site—alongside safety and other essential postings—helps to communicate to workers and subcontractors that the site values worker mental health. Given that workers and contractors frequently change jobs and sites over time, this communication is valuable both to current workers (eg, affirming their ongoing participation in the program) and those newly arriving (who may or may not have participated in MATES programs at previous sites). For contractors (primary and otherwise) and unions in the sector, MATES accreditation demonstrates commitment to workplace mental health that goes beyond compliance with OH&S and other legal requirements, representing good corporate citizenship or social responsibility. Positively constructive peer pressure for accreditation could also be created by accreditation of industry-leading sites and projects. Accreditation criteria can also promote program uptake by inclusion as a condition of government or other contracts. As of 2024, MATES has accredited over 414 construction sites in Australia and New Zealand.

It is important to recognize that “accreditation” is not an end in itself and further, there is a risk that accreditation processes can become a “tick box” exercise. This can be mitigated by ensuring a sound and transparent basis for accreditation criteria, that in turn will ensure that accreditation is meaningful. Articulation and sharing of the basis for MATES’ accreditation criteria are among the purposes of this article.

Arising from an internal review and refinement of the MATES accreditation criteria in 2025, and with the purpose of informing evolving policy and practice in the broader field of workplace mental health and suicide prevention, this article presents the following:

- The purpose and history of MATES’ accreditation criteria;
- The rationale and evidence underpinning the MATES accreditation criteria;
- The evolving practice of accreditation in the MATES context

The article outlines the methods used in conducting the internal review, followed by the four current accreditation criteria, an explanatory section on each criterion, the applicability of the criteria to other MATES programs, a description of the accreditation process as currently practiced, consideration of accreditation in light of the continuing evolution MATES program (eg, integration of new program elements), and a summary and acknowledgement of the limitations of accreditation in achieving MATES's goal of reducing suicide rates in the construction and related sectors.

## Methods

This article is based on the outcomes of several stages of review and consultation. First, a review of all available MATES documents on accreditation (historical and current) was conducted by the first 2 authors. This was followed by consultation with MATES staff about the history of accreditation in the MATES organization, the rationale underpinning the criteria, current accreditation practice, and potential refinements. On the basis of this review and consultation, a document summarizing the provisional findings of the review, integrated with relevant principles, theory, and empirical evidence from the published literature drafted by the first 2 authors. This was reviewed by the MATES Executive team (leaders and senior staff of the MATES federation members who are all MATES employees [authors 3–9]), leading to a second draft of the document. This was reviewed by the MATES Executive team before they participated in an in-person workshop to finalize the review and accreditation criteria update.

It is important to note that this document arose out of an internal review and was not conducted as research. Thus, there was no formal protocol or ethical review of the process. Because we thought that the outcomes of this process (*post hoc*) would be of interest to other workplace mental health and suicide prevention practitioners, we thought there would be value in sharing these findings publicly in a context such as NEW SOLUTIONS’ *Comment and Controversy* section.

## Mates Accreditation Criteria

The MATES site accreditation criteria were first developed in the early years of the program. Initially, accreditation included the first three criteria, with the fourth added subsequently. The criteria were reviewed and refined most recently in 2025. These are the refined accreditation criteria, italicized to denote that this is the language of MATES internal policy (the terms “Connector” and “Applied Suicide Intervention Skills Training (ASIST)” are defined in the following sections), following the review described in the Methods sections above:

*Work sites are eligible to achieve MATES “Accredited” status. To be awarded “Accredited” status, the site must meet the following criteria:*

1. *All the available workforce on a work site, including administration staff, must be offered General Awareness Training (GAT).*
2. *A process must be in place to ensure that at any point in time following initial GAT training, at least 80% of the workforce is GAT trained.*
3. *Workers across the work site must have a line of sight to a Connector they would reasonably identify with. It is suggested that a 1:20 ratio is maintained.*
4. *The work site must have a process for access to an ASIST-trained person during operational hours. Where possible, these ASIST-trained persons should be on site.*

*These accreditation principles will apply across all MATES entities and programs. Accreditation will be assessed by relevant Field staff, and a local process of moderation will be established. Moderation means a peer review process striving for consistency and transparency in the accreditation process.*

## Rationale for Accreditation Criteria

*Criterion 1:*

- *All the available workforce on a work site, including administration staff, must be offered GAT.*

This criterion is justified because GAT is a *universal preventive intervention* within the construction industry. The prevention and early treatment spectrum for mental health/illness describes three types of interventions: universal, selective, and indicated.^10,11^ *Universal* interventions are provided to all workers, independent of their risk or mental health status. *Selective* interventions are aimed at workers who are at an increased risk for mental illness due to the nature of their work or for other reasons, and *indicated* interventions are directed at distressed workers with elevated mental illness symptoms. The prevention and early treatment spectrum is essentially synonymous with a “public health approach” to suicide prevention.^12^

GAT aims to engage workers in suicide prevention efforts through a community organizing outrage/hope/action strategy.^13,14^ GAT aims to raise all workers’ awareness that (1) the suicide rate in construction is higher in construction than in the rest of the working population (“outrage”), (2) suicide is preventable (“hope”), and (3) there are actions you can take whether you are at risk or not (“action”). Those actions include seeking help from a Connector if one is distressed, offering help to coworkers, and encouraging mates in distress to talk to a Connector. It is crucial that all workers on-site receive this training to create a suicide-aware and supportive culture on-site and to communicate that all workers can participate in reducing suicide risk, whether they stop at GAT training, become Connector volunteers, or become ASIST workers. By positively affecting the culture of a work site, the MATES program can promote social support, connection, and cohesion, which in turn can promote health and well-being, even among those who do not experience mental health problems. Hence, MATES can contribute to both the promotion of mental well-being and the prevention of mental health problems.^13^ Finally, GAT sessions convey antistigma messaging universally, aiming to facilitate help-offering and help-seeking.^13^

The community organizing strategy underpinning the MATES program is reinforced by the industry basis of the program.^14^ The MATES program arose through an agreement between labor and management in the sector that something needed to be done about the elevated suicide rates, and that it needed to be done from within *by the construction industry*. The universal aspect of GAT further reflects this collective responsibility for reducing suicide in construction.

Another objective of GAT training is to make people aware of what Connectors are (volunteers trained in how to “connect” people to help, as detailed further below) and how to call on them. Given that only those who are aware of Connectors would use them, and anyone can experience distress, it is therefore necessary that everyone on site is GAT trained in order to be aware of Connectors and, therefore, able to contact a Connector when needed.

Finally, the MATES universal GAT training approach is consistent with emerging evidence of the potential for intentionally modifying social structures to reduce social isolation and help prevent suicide.^15^

**Figure 1.**
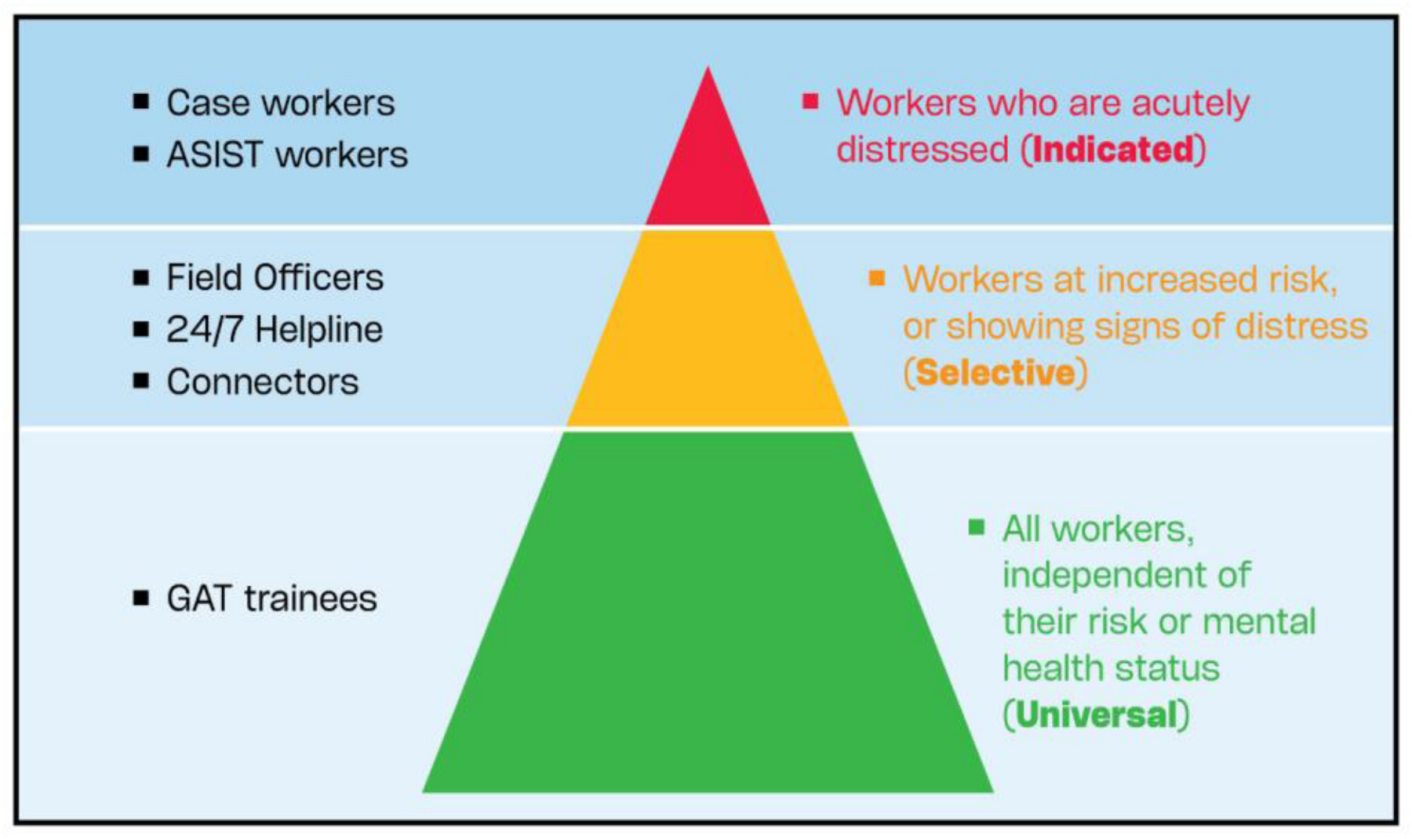
MATES’ network of safety: key program elements on the left and worker groups and corresponding prevention and early treatment strategies on the right (universal/selective/indicated), with the triangle representing the construction worker population.

On the basis of these considerations, it is clear that GAT training must be available to all workers on a site, that is, GAT training must be *universal*. This is illustrated in Figure 1.

While GAT is universal, other elements of the MATES program are *selective* or *indicated* (Figure 1). An example of a *selective* intervention is a Connector checking in on a worker either due to the worker “not seeming to be his/her/themselves” or as a result of concern expressed to the Connector by a coworker. An example of an *indicated* intervention would be a suicidal crisis intervention by an ASIST worker and the case management that would follow.

Evaluation research on MATES programs has shown implementation-associated reductions in stigma and improvements in suicide literacy, help-seeking intentions, and help sought among GAT trainees.^16,17^ These findings are consistent with those of a recent meta-review of universal, selective, and indicated interventions in workplace settings, which showed small to moderate beneficial effects across the various outcomes of symptoms of mental health conditions, positive mental health, quality of life, work-related outcomes, substance use, and suicide-related outcomes.^11^

*Criterion 2:*

- *A process must be in place to ensure that at any point in time following initial GAT training, at least 80% of the workforce is GAT trained*

It is important to note that “universal” refers to all workers on a site, including blue-collar and white-collar, principal and subcontractor workers, and gig, casual, fixed-term, or permanent workers (more on this below). While 100% of workers on-site is the ideal, MATES is also cognizant of feasibility challenges. Maintaining 100% of workers trained will be difficult in the face of ongoing turnover of workers, changing contractors over time, etc*.* These are normal conditions in the building and construction sector. For MATES, there are also feasibility challenges in terms of the frequency with which MATES field staff can provide GAT training.

MATES has set 80% as the reasonable minimum to ensure that most workers on-site are GAT trained, approximating universal intervention, and thus going most of the way toward creating a suicide-aware site.

*Criterion 3:*

- *Workers across the work site must have a line of sight to a Connector they would reasonably identify with. It is suggested that a 1:20 ratio is maintained.*

Connectors are volunteers recruited through GAT training sessions who undergo a half-day adaptation of the *LivingWorks Safe Talk* training. The intent of having 1:20 workers on-site, Connector-trained, is that a Connector would be readily identifiable and accessible to any worker on-site who may be in distress.

The 1:20 ratio (5%) is a simple, pragmatic criterion that originally came from Living Works,^18^ a global leader in suicide prevention skills training. It is based loosely on a ∼5% prevalence of suicidal ideation in the general adult population internationally. In the construction industry, it is ∼7.5% as an international average^19^; and a recent study estimated the 12-month prevalence of suicidal ideation in the West Australian construction industry at 10.7% compared to a national average of 3.3% among the general Australian adult population.^20^ The logic for this is that if 1 in 20 have thoughts about suicide, most of the others are aware of the signs, and a *Living Works Safe Talk*-trained Connector is at hand, then not only will the person with suicidal ideation be recognized, but that person is more likely to receive help offered by coworkers, referral to a Connector, and/or direct intervention from a Connector or an ASIST worker. Hence, the 1:20 ratio is not a rigid criterion, but more of a benchmark. Site-specific circumstances might justify higher or lower ratios. For example, if groups of 10 to 15 are working in separate spaces (eg, floors of a high-rise building), then 1:10 to 1:15 ratios could be justified. Similarly, if those groups were 25 to 30, then 1:25 to 1:30 could be justified. In either case, the aim is to provide a “line of sight” to a Connector for all workers.

Visual cues to enhance identifiability of Connectors include green MATES Connector hardhat stickers and a Connector Board on site with names and photos. Accessibility would be further facilitated by everyone on-site having a Connector at their peer level (eg, blue-collar worker to worker, manager to manager, or admin to admin). This minimizes potential occupational power dynamics. It is informed by previous evaluation research among GAT trainees, indicating that *the least* likely person a worker would seek help from due to mental difficulties is their supervisor or manager.^16^ In a related context, the guidelines for Mental Health First Aid include avoiding the administration of First Aid by supervisors to supervisees to protect against the possibility that information shared could have adverse consequences (eg, affecting job retention or promotion prospects).^21^

The 1:20 ratio also aims to provide a distribution of Connectors on-site by proximal physical location and/or by nature of work (eg, office versus job site, manual vs technical work). Another consideration is the promotion of diversity among Connectors. For example, the proportions of females and migrants, as well as the diversity of cultural, linguistic, and/or occupational backgrounds on-site, should be reflected in the Connector group. In short, the more options a distressed worker has for seeking help, the more likely they’ll be able to talk to someone they identify with or feel comfortable with, and the more likely they will be able to seek help at the level of discretion, confidentiality, and safety desired.

The 1:20 level is intended as a pragmatic benchmark toward achieving a Connector group that reflects the composition of the site's workforce. Thus, a formal assessment of the representativeness of a given Connector group is not articulated in the Accreditation criteria. However, it is informally integrated into the Accreditation process, which involves Field Officers (paid MATES professional staff) and other MATES staff. Representativeness of the Connector group should be included in the presentation made by the Field Officer responsible for the site under evaluation to other MATES Field Officers, the Operations Manager, and other senior MATES staff. In summary, Accreditation is determined at the group level rather than at the discretion of a single Field Officer.

Finally, as noted above, a readily identifiable and accessible Connector group provides *selective* intervention capacity in the *universal, selective, indicated* spectrum referred to above.^1^

*Criterion 4:*

- *The work site must have a process to access an ASIST-trained person during operational hours. Where possible, these ASIST-trained persons should be on site.*

ASIST is a 2-day *Living Works* course adapted to the MATES context to train workers in how to intervene in a suicidal crisis. Because suicidal crises can occur at any time, prompt access to an ASIST worker is essential. ASIST-trained workers are identifiable by a blue hard hat sticker. Access to an ASIST worker, along with the toll-free helpline and case management, completes the *universal/selective/indicated* preventive intervention spectrum^10^ and provides the foundation for the *network of safety* that the MATES program aims to provide (Figure 1).

All MATES Field Officers are ASIST trained and can provide this function for the sites they serve when the sites are located in major metropolitan areas where MATES offices or Field Officers are located. Accreditation requires an on-site ASIST worker only for locations in regional and remote areas, so as to avoid delayed access to an ASIST worker in the event of a suicidal crisis.

We have changed the previous language of “ASIST-trained resource” (in previous versions of accreditation criteria) to “ASIST-trained person” to make it more concrete. Importantly, an “ASIST-trained person” could be a MATES Field Officer, an ASIST-trained MATES volunteer, or someone with ASIST training acquired through other programs, including members of the local community who could be called upon. While having an ASIST-trained person on-site during all operational hours is the ideal, this flexibility is required in particular for small sites.

## Accreditation Criteria Across the Mates Family of Programs

The current Accreditation criteria were created for the construction sector, before MATES expanded into the other predominantly male blue-collar sectors of mining, energy, and manufacturing. The same Accreditation criteria apply in all industrial sectors in which MATES operates—currently Mining, Energy, and Manufacturing. This is because the relevant principles expressed above are applicable across contexts, including the full spectrum of workplace and community contexts. For example, the universal/selective/indicated spectrum of mental health interventions does not vary by context, nor do the *Living Works* training recommendations vary by community context. The *Living Works* programs for creating suicide-safer communities are generalizable across communities, and hence across various industrial sectors as well.

In addition, the same set of Accreditation criteria will apply across the MATES federation, presented by its members in various Australian states and territories as well as in New Zealand.

## The Accreditation Process

Each participating site or workplace has a responsible Field Officer (paid MATES staff member) who looks after that site. Once the responsible Field Officer believes the site has met the accreditation criteria, the case for accreditation is presented to a group of MATES peers—usually consisting of fellow Field Officers, the MATES Operations Manager, and other senior MATES staff.

Information on each of the criteria is presented, and the basis of the information presented is reviewed, responding to questions such as:

- *Criterion 1*: Have all of the workers on site been offered GAT training? Has this included blue and white collar workers, all contractors, etc? How was this determined?
- *Criterion 2*: Does the site have a process for insuring that at least 80% of workers on are GAT-trained at present and will continue to be going forward? When might the site need additional GAT trainings to cover new workers, contractors, *etc*, coming on site over time? Is this addressed in the site's process for maintaining ≥ 80%?
- *Criterion 3*: On the basis of what evidence does the site claim to have provided Connector training to at least one in 20 workers? Are there site-specific conditions that justify a different ratio? How well does the group of trained Connectors on site represents the site's population, including different occupational groups, trades, contractors, genders, and varying cultural and linguistic backgrounds? When might the site need additional Connector trainings to address turnover, and the arrival on-site of new workers and contractors, *etc*?
- *Criterion 4*: What is the site's strategy or process for meeting the need for an ASIST-trained person to be accessible during all operational hours? If there is not an ASIST-trained person on site, can a local ASIST-trained person be accessed in a timely fashion if and when the need arises? If the site does not have one or more ASIST-trained persons on site, is the site interested in arranging training for one or more volunteers?

This collective determination of accreditation is designed to ensure consistency and transparency in the process and its administration across the organization, and to prevent bias or favoritism.

MATES is currently considering how to acknowledge GAT and other training from sites other than the site under consideration for accreditation. Because MATES is an industry-based program, individuals moving between sites, projects, or organizations will often have had previous MATES training. It is acknowledged that recent training should count regardless of where it was obtained. Pending questions under consideration include:

- In relation to inclusion of training obtained at a different site (and the need for refresher training), for what period should GAT, Connector or ASIST training be considered “current”? This is likely to be at least 1 year, possibly longer.
- How should prior training be demonstrated or documented? This will be explored through the MATES database and possibly a MATES smartphone app that was recently developed.

## New and Emerging MATES Program Elements

The Accreditation criteria to date address the core elements of the MATES program that have been integral since its inception in 2008. The MATES program has continued to evolve, and new program elements have been added to address additionally identified needs, as outlined below.

In response to alarming findings about the high prevalence of bullying, distress and suicidal ideation among apprentices, MATES developed a threefold training intervention program to address bullying, mental health and suicidality of apprentices in the construction industry. The program consists of Awareness Training for Supervisors, Resilience Skills for Apprentices, and the Apprentice Awareness Toolbox for the overall workforce. The half-day Apprentice training covers workplace bullying, suicide and mental health literacy, financial management, gambling, alcohol and other drugs, and workplace rights and responsibilities. The half-day Awareness Training for Supervisors addresses the obligations of leaders (leading hands, supervisors, managers, etc) in relation to workplace bullying, harassment, and discrimination, and how to respond to these and other challenging situations. The Toolbox training for the general workforce is offered as a standup 15-min session. It emphasizes the distinction between bullying and banter and the need for collective intervention when an apprentice is bullied (strong emphasis on “don’t be a bystander”). MATES has been delivering the ongoing training sessions for this multilevel program in Queensland since 2022. A recent pre–post evaluation involving 95 apprentices (Resilience Skills for Apprentices), 89 supervisors (Awareness Training for Supervisors), and 743 general trade workers (Apprentice Awareness Toolbox) has shown evidence of effectiveness in improving proximal outcomes of knowledge, confidence, and awareness in relation to issues around bullying, as well as how to access and provide help.^22^

MATES RESPOND training extends the skills of trained Connectors or ASIST workers, equipping them to provide guidance after a death or Critical Incident while waiting for further support. The program uses 3 key elements based on a synthesis of previous studies: connecting with the event, understanding the event, and providing support to workers and to the site.^23^

An open access tool entitled “People at Work” (P@W) is provided by the Australian government for conducting workplace psychosocial risk assessment. MATES commissioned an adaptation of the P@W survey for the construction sector,^24^ referring to the tool as P@W-CON. A valuable case study illustrates its application in psychosocial hazard risk assessment, management, and evaluation in a civil construction project in Queensland.^25^

The Blueprint for Better Mental Health and Suicide Prevention^26^ was launched by MATES in Construction in 2018 with the support and participation of Beyond Blue (the Australian national depression and suicide prevention initiative), the Construction Forestry Mining and Energy Union, National Fire Industry Association, Master Plumbers Australia, Master Builders Australia, and Queensland Major Contractors Association. Informed by the “integrated approach” to workplace mental health,^27,28^ the Blueprint supports workplace efforts to extend the MATES program in the following priority areas: (1) reducing the harmful impacts of work, (2) providing mental health and suicide prevention literacy, (3) facilitating early intervention and treatment, (4) providing return-to-work and ongoing support, and (5) promoting work's positive impacts on mental health. While the MATES program originated in its early years with a reactive focus (responding to workers in distress), it is progressively developing more “upstream” preventive elements, including addressing working conditions that can be harmful to mental health.

These additional program elements are not currently assessed in the foundational accreditation criteria described above. In the future, MATES may consider progressive or higher levels of Accreditation to recognize the implementation of increasingly comprehensive workplace mental health programs, including the above-described recent additions to the MATES program offerings.

## Summary and Limitations of Accreditation

The MATES accreditation criteria are based on meeting training requirements because these are readily observable benchmarks. The MATES program, however, is far more than a set of training programs. As outlined above, the GAT, Connector, and ASIST criteria are designed to provide the foundation for a network of suicide safety on-site. The full network is realized when the participation of workers, Connectors, and ASIST-trained persons connect workers in need to help—through Field Officers, the 1-800 helpline, and the MATES case management system. The MATES approach is consistent with emerging evidence of the potential for intentionally modifying social structures to help prevent suicide by reducing isolation.^15^ In addition to responding to workers in distress, the MATES program can build workplace social connection and support, helping to promote well-being and prevent mental health problems across the workplace population.^2,14^ Further, the program requires the cooperation and participation of labor and management, which is optimized by MATES’ status as an independent industry-based charity, jointly controlled by labor and management.^6,14^ Training and accreditation provide a foundation, but participation of all workplace stakeholders and the MATES organization is required for the MATES program and the network of suicide safety to be fully enabled.

The extent to which the MATES justification for, and approach to, accreditation is generalizable to other contexts is not known, but warrants further evaluation research (eg, to determine the extent to which accreditation enhances reach and implementation). However, the underlying principles and rationale for accreditation are adaptable to other workplace and broader community contexts because they are based on evidenced-based theories in public health and mental health (eg, the spectrum of mental health interventions). We do not claim that the MATES approach is the best or only approach to accreditation. Moreover, we note that accreditation is only one strategy for promoting the dissemination and reach of a program while maintaining minimum standards for implementation. For related workplace mental health and suicide prevention programs, program leaders will need to consider the social, political, and economic contexts in which their programs operate in order to devise an optimal strategy for promoting reach and implementation. For example, a pilot project, currently underway across six construction sites in North America, aims to determine whether and how the MATES program might be adapted to that context, which is very different from that of Australia. At this stage, accreditation is not part of the North American pilot program.

Whether it will be in the future is yet to be determined. This article is offered to other practitioners so that they may take what they find useful from the MATES experience and adapt it to their particular contexts, with the ultimate aim of improving policy and practice toward the reduction of suicide in construction and other predominantly male blue-collar workplace contexts.
